# Respiratory arousal threshold among patients with isolated sleep apnea and with comorbid insomnia (COMISA)

**DOI:** 10.1038/s41598-023-34002-4

**Published:** 2023-05-11

**Authors:** Marcela Yanagimori, Mariana D. Fernandes, Michelle L. Garcia, Paula G. Scudeller, Carlos R. R. Carvalho, Bradley Edwards, Geraldo Lorenzi-Filho, Pedro R. Genta

**Affiliations:** 1 Laboratorio do Sono, LIM 63, Divisao de Pneumologia, Instituto do Coracao (InCor), Hospital das Clinicas HCFMUSP, Faculdade de Medicina, Universidade de Sao Paulo, Sao Paulo, SP Brazil; 2 Sleep and Circadian Medicine Laboratory, Department of Physiology and School of Psychological Sciences, Faculty of Medicine, Nursing and Health Sciences, Monash University, Melbourne, VIC Australia

**Keywords:** Psychiatric disorders, Respiratory tract diseases, Diseases

## Abstract

Insomnia and obstructive sleep apnea (OSA) are common sleep disorders and frequently coexist (COMISA). Arousals from sleep may be a common link explaining the frequent comorbidity of both disorders. Respiratory arousal threshold (AT) is a physiologic measurement of the level of respiratory effort to trigger an arousal from sleep. The impact of COMISA on AT is not known. We hypothesized that a low AT is more common among COMISA than among patients with OSA without insomnia. Participants referred for OSA diagnosis underwent a type 3 sleep study and answered the insomnia severity index (ISI) questionnaire and the Epworth sleepiness scale. Participants with an ISI score ≥ 15 were defined as having insomnia. Sleep apnea was defined as an apnea hypopnea index (AHI) ≥ 15 events/h. Low AT was determined using a previously validated score based on 3 polysomnography variables (AHI, nadir SpO_2_ and the frequency of hypopneas). OSA-only (n = 51) and COMISA (n = 52) participants had similar age (61[52–68] vs 60[53–65] years), body-mass index (31.3[27.7–36.2] vs 32.2[29.5–38.3] kg/m^2^) and OSA severity (40.2[27.5–60] vs 37.55[27.9–65.2] events/h): all *p* = NS. OSA-only group had significantly more males than the COMISA group (58% vs 33%, *p* = 0.013. The proportion of participants with a low AT among OSA-only and COMISA groups was similar (29 vs 33%, *p* = NS). The similar proportion of low AT among COMISA and patients with OSA suggests that the respiratory arousal threshold may not be related to the increased arousability of insomnia.

## Introduction

Obstructive sleep apnea and insomnia are highly prevalent sleep disorders that are associated with impaired daytime functioning and reduced quality of life^1^. The comorbidity of insomnia and sleep apnea (COMISA) is associated to poorer health outcomes^2^. Arousals from sleep are common in both insomnia and OSA and may be a common link explaining the frequent comorbidity of both disorders.

The respiratory arousal threshold (AT) is defined as the level of ventilatory drive that triggers an arousal from sleep at the termination of a respiratory event^3^. A low AT has been implicated in the pathogenesis of OSA and may affect one-third of moderate to severe OSA patients^4^. Patients with a low AT typically have respiratory events that terminate early, preventing the opportunity for ventilatory drive to build up and restore pharyngeal patency during sleep and promoting sleep fragmentation. Arousability may describe a particular phenotypical trait of insomnia patients^5^. Arousability in these patients can be defined as the physiological, cognitive, emotional, and behavioral responsiveness of individuals to particular variations in environmental stressful conditions^6^ and is associated with insomnia severity as measured by the Insomnia Severity Index (ISI)^6^.

Insomnia subtypes are associated with co-existing disorders such as depression, anxiety and sleep apnea^7^. Middle insomnia is the most prevalent subtype in COMISA subjects, possibly due to frequent arousals at the end of respiratory events^1,8^. The arousal threshold has not been studied according to the different insomnia subtypes.

Understanding the relationship between respiratory AT and arousability can shed light to the understanding of the pathophysiology implicated in COMISA and allow targeting more specific treatments. For instance, hypnotics are commonly used to treat insomnia^9^. Hypnotics aiming to increase the arousal threshold have also been tried as a treatment for OSA, resulting in OSA improvement for some individuals^10–12^. The purpose of the present study is to assess the AT among participants with COMISA and those with OSA only. We hypothesized that a low AT is more common among COMISA than among OSA patients without insomnia.

## Participants and methods

### Participants

Participants included in this study participated in a larger prospective study between September 2019 and February 2020 that addressed clinical characteristics of OSA among patients undergoing a sleep study for suspected OSA. After written informed consent was obtained, all participants answered standardized questionnaires about insomnia and daytime sleepiness. Demographic and anthropometric data included age, gender, BMI, neck circumference, comorbidities and sleep questionnaires.

### Questionnaires

Insomnia was characterized using the Insomnia Severity Index (ISI) questionnaire. The ISI questionnaire assesses symptoms and severity of insomnia using 7 standardized questions. Participants with an ISI score ≥ 15 were defined as having insomnia^13^. Initial, middle and late insomnia subtypes were characterized using the answers of the three ISI questions assessing difficulty falling or staying asleep and waking up too early. In these 3 questions, participants checked one of the five responses (none, mild, moderate, severe and very severe). Severe and very severe answers were used to define insomnia subtypes. ISI nocturnal sub-score was calculated by the sum of first three items from the ISI questionnaire.

Daytime sleepiness was evaluated with the Epworth Sleepiness Scale (ESS). The ESS assesses the likelihood of falling asleep during eight common daily situations, with higher scores indicating greater sleepiness. An ESS ≥ 11 was used to define excessive daytime sleepiness^14^.

### Sleep studies

Sleep studies were performed at home using a type 3 monitor (Apnealink Plus, Resmed, San Diego, CA). This device has an oximeter, a thoracoabdominal effort belt and nasal cannula. All studies were reviewed by a single investigator, according to the American Academy of Sleep Medicine (AASM) Manual for Scoring Sleep and Associated events. The minimum duration of respiratory events was 10 s(s). Apneas were defined as a cessation of airflow for 10 s. Hypopneas were defined as a reduction of the airflow signal amplitude ≥ 30%, associated with a 3% oxygen desaturation. The AHI was calculated as the number of apneas and hypopneas divided by the recording duration in hours. OSA was defined as an AHI ≥ 15 events/h.

### Arousal threshold

Low AT was determined using a previously validated score based on 3 polysomnography variables that considers one point for each of the following 3 criteria: AHI < 30 events/h, a nadir SpO_2_ > 82.5%, and the proportion of hypopneas/sum of apneas and hypopneas > 58.3%^3^. Patients were classified as having low AT based if the score was ≥ 2.

### Eligible group

From the database of 998 patients, 920 answered the ISI questionnaire. Using insomnia (ISI ≥ 15) and OSA (AHI ≥ 15 events/h) criteria, participants were classified into four groups: (1) none (231 participants); (2) OSA-only (261 participants); (3) insomnia only (199 participants) and; (4) COMISA (OSA and insomnia) (229 participants). Since we did not have a preliminary study to base the expected difference in arousal threshold, we studied a random sample of 103 participants out of the entire group of 490 participants with COMISA and OSA-only.

### Statistical analysis

The normality of the distribution of continuous variables was assessed with the Kolmogorov–Smirnov test. Continuous variables were described as mean ± standard deviation or median [25th–75th percentiles]. Categorical variables were presented as whole numbers and percentages. Differences between variables were compared using the chi-square test, Student´s unpaired t- or Mann–Whitney tests. Analyses were performed with IBM SPSS Statistics 20 (Chicago, USA).

All methods were carried out in accordance with relevant guidelines and regulations and all protocols were approved by Comissão de Ética e Pesquisa (CEP) from FMUSP.

## Results

Fifty-one participants from the OSA-only group and 52 participants from the COMISA group were randomly selected from the larger database of 490 participants. Anthropometric and clinical characteristics of the study population (n = 103) are shown in Table 1 and did not differ from the larger database (data not shown). Patients with OSA-only were predominantly male, while those with COMISA were predominantly female. OSA severity was similar between groups. The proportion of participants with a low AT was similar between OSA-only and COMISA groups. As expected, ISI was lower among OSA-only as compared to COMISA participants. Sensitivity analysis considering each gender separately did not affect the results observed when the whole group of participants were analyzed (data not shown).

**Table 1:** Anthropometric and clinical characteristics of study population.

	OSA-only (n = 51)	COMISA (n = 52)		*p*	
Anthropometric					
Male, n (%)	34 (67)		22 (42)		0.01
Age, years	61 [52–68]		60 [53–65]		0.23
BMI (kg/m^2^)	31.3 [27.7–36.2]		32.2 [29.5–38.3]		0.36
Comorbid diseases					
Hypertension, n (%)	25 (49)		29 (55.7)		0.49
Arrhythmia, n (%)	10 (19.6)		11 (21.1)		0.84
COPD, n (%)	6 (11.7)		7 (13.4)		0.79
Diabetes, n (%)	14 (27.4)		14 (26.9)		0.95
Dyslipidemia, n (%)	23 (45)		23 (44.2)		0.92
Gout, n (%)	1 (1.9)		4 (7.6)		0.17
Heart attack, n (%)	8 (15.6)		3 (5.7)		0.10
Stroke, n (%)	3 (5.8)		2 (3.8)		0.63
GERD, n (%)	10 (19.6)		19 (36.5)		0.05
Depression, n (%)	3 (5.8)		10 (19.2)		0.04
Questionnaires					
ESS	11.2 ± 5.9		13.2 ± 5.7		0.08
ISI	9.2 ± 3.1		19.1 ± 3.1		< 0.001
PSG variables					
AHI	40.2 [27.5–60]		37.55 [27.9–65.2]		0.79
Nadir SpO_2_	75.2 ± 5.3		75.7 ± 5.1		0.62
Low AT and variables					
Low AT, n (%)	15 (29)		17 (33)		0.71
AHI < 30, n (%)	16 (31.3)		15 (28.8)		0.78
Lnadir SpO_2_ > 82.5%, n (%)	6 (11.7)		8 (15.3)		0.59
Fhypopneas > 58.3%, n (%)	31 (60.7)		35 (67.3)		0.49

Data is shown as mean ± standard deviation or median (25–75%).

*BMI* body-mass index, *COPD* chronic obstructive pulmonary disease, *GERD* gastroesophageal reflux disease, *ESS* Epworth sleepiness scale, Fhypopneas proportion of hypopneas/respiratory events, *ISI* Insomnia Severity Index, *AT* arousal threshold.

Characteristics of low AT and high AT groups are shown in Table 2. Compared with the high AT group, patients with low AT were composed of a higher percentage of women, were less obese and had lower AHI.

**Table 2:** Clinical characteristics of participants with Low and High AT.

	Low AT (n = 32)	High AT (n = 71)	*p*
Male, n (%)	11 (32)	26 (64)	0.01
Age, years	59 [52–66]	61 [53–68]	0.66
BMI (kg/m^2^)	32 ± 5	34 ± 7	0.03
AHI, events/h	23 [19–24]	53 [38–76]	< 0.001
ISI	15 ± 6	14 ± 6	0.18

Data is shown as mean ± standard deviation or median (25–75%).

*BMI* body-mass index, *ISI* Insomnia Severity Index, *AT* arousal threshold.

In patients with COMISA, ISI scores were similar among participants with a low AT 19[18–22] (n = 35) and participants with a high AT 18[16–21] (n = 17) (*p* = 0.34). Initial, middle and late insomnia was observed among 38.5, 65.4, 32.7% of COMISA participants. The proportion of low and high AT was similar among each insomnia subtype (initial, middle and terminal). ISI nocturnal sub-score was also similar between low and high AT (Table 3). The presence of comorbidities was similar between groups, except for depression, which was more prevalent among COMISA participants (*p* = 0.04).

**Table 3:** Clinical characteristics of participants with COMISA according to a Low and High AT.

COMISA	Low AT (n = 17)	High AT (n = 35)	*p*
Male, n (%)	3 (18)	19 (54)	0.01
Age, years	60 [53–66]	57 [49–65]	0.44
BMI (kg/m^2^)	32 ± 6	34 ± 6	0.18
AHI, events/h	23 [19–29]	56 [37–76]	< 0.001
ISI	19 [18–22]	18 [16–21]	0.16
Initial insomnia, n (%)	6 (35)	14 (40)	0.74
Middle insomnia, n (%)	12 (71)	22 (63)	0.58
Terminal insomnia, n (%)	7 (41)	10 (28)	0.36
ISI nocturnal sub-score	7 ± 2	6 ± 2	0.26

Data is shown as mean ± standard deviation or median (25–75%).

*BMI* body-mass index, *ISI* Insomnia Severity Index, *AT* arousal threshold.

## Discussion

The major finding of our study was that insomnia did not impact arousal threshold among OSA patients as COMISA patients and OSA-only patients had a similar proportion of low AT. Insomnia severity was also similar among COMISA patients with or without a low AT. These findings suggest that the mechanisms leading to respiratory arousals are different than spontaneous arousals experienced by insomnia patients.

Arousal threshold can be defined as the magnitude of ventilatory drive in response to a respiratory stimulus terminating with an arousal^15^. The concept of AT was defined by the observation that negative inspiratory pleural pressure during the breaths preceding arousal were similar, whether stimulated by added inspiratory resistive load, hypoxia, or hypercapnia^15^. This finding supported the hypothesis that arousal from sleep resulting from the stimulation of ventilation is due to the increase in ventilatory drive. Participants with a low AT have been referred as “light sleepers” or “easily arousable”, suggesting that AT would be a marker of arousability regardless of respiratory effort^12,16,17^. Conditioned arousals to airway occlusion have been suggested as an alternative mechanism leading to arousals in insomnia patients and may be associated with higher arousability^12^. In the present study, the proportion of participants with a low AT was similar among OSA patients with and without comorbid insomnia. In addition, insomnia severity was also similar among participants with a low and high AT. A previous study addressed AT among OSA patients with insomnia and post-traumatic stress disorder (PTSD)^4^. Although insomnia was associated with low AT, only male OSA veterans with PTSD were studied^4^.Two recent abstracts addressed the arousal threshold in COMISA subjects in smaller populations. In one study, no relationship between ISI and AT in the whole group that included OSA-only and COMISA subjects (n = 46) was observed^18^. However, an inverse association between ISI and AT in the subgroup of COMISA subjects (n = 27) was reported^18^. OSA severity and AT among OSA and COMISA groups were not reported or compared^18^. In another abstract, AT was lower among COMISA subjects^19^. However, COMISA subjects tended to have milder OSA, which may bias AT comparison since patients with a low AT tend to have milder OSA severity^17^. Some studies have shown that a low AT is associated with poor CPAP adherence^16,17,20,21^.. However, this association may also be biased by lower OSA severity, which has been shown to be a strong predictor of CPAP adherence^22,23^. For instance, the difference in AHI among participants with a low and high AT in a previous study (17 and 48 events/h, respectively)^17^ is similar to the findings of the present study (23 and 53 events/h, low and high AT respectively). Therefore, milder OSA severity may bias the comparison of AT and CPAP adherence between OSA-only and COMISA subjects.

Middle insomnia is the most common insomnia subtype among COMISA participants^1,8^. In the present study, middle insomnia was also the most common insomnia subtype. Although insomnia subtype comparison was underpowered, participants with isolated middle insomnia had a similar proportion of low AT as compared to other insomnia subtypes. Arousability is a trait of insomnia that can be defined as the physiological, cognitive, emotional, and behavioral responsiveness of individuals to particular variations in environmental conditions, particularly stressful ones^6^. Arousability can be determined using self-reported instruments^24^ and has been associated with insomnia severity as measured by the ISI^6,25^. The similar proportion of low AT between COMISA and OSA-only participants and among isolated middle and other insomnia subtypes suggest that the higher arousability found among insomnia participants is a different phenomenon than the ventilatoty effort-related arousal threshold.

Our findings agree with previous studies that compared clinical characteristics of low and high arousal threshold groups. In the present study, low AT patients had a lower BMI, were predominantly female and had a lower AHI^17,26,27^. Another study showed that normal body weight was a poor predictor of CPAP adherence among participants with a low AT^16^. Nevertheless, the major predictor of CPAP use was the interaction between obesity and low AT, not any of these variables alone^16^. Participants with OSA may increase their AT during the course of the disease, tolerating increasingly longer obstructive events as the disease evolves. After a period of CPAP treatment, AT tends to decrease^28^, reinforcing the concept that a higher AT may be a consequence of OSA severity.

We should point recognizable limitations of the present study. First, low AT determination was based on an algorithm and not based on the invasive quantitative assessment conducted in specialized physiologic laboratory. Second, the algorithm used for calculating the arousal threshold has been validated for OSA patients, but not specifically for COMISA subjects. However, this approach allowed us to analyze a reasonable number of subjects. Third, we used a type 3 monitor, which may underestimate OSA severity and impact the classification of AT. Nevertheless, the type of sleep monitor used would affect both groups equally and would unlikely affect the final results. Fourth, the sample size was not determined a priori due to the lack of previous studies to derive information. Fifth we used ISI to define insomnia, instead of the diagnostic and statistical manual of mental disorders criteria (DSM). However, ISI has a high accuracy to detect insomnia and has the advantage of being able to determine insomnia severity^29^. Finally, one night of monitoring can lead to misclassification of disease severity in patients with mild and moderate OSA^30,31^. However, most included subjects had severe OSA.

In summary, a similar proportion of low AT among OSA-only and COMISA patients. Our findings suggest that a low AT is not a characteristic of COMISA patients.

## Data Availability

The datasets generated during and/or analyzed during the current study are available from the corresponding author on reasonable request.
